# Adequate application of chicken manure could relieve the damage caused by Cd to *E. breviscapus* plants

**DOI:** 10.3389/fpls.2025.1539907

**Published:** 2025-09-04

**Authors:** Lijie Jia, Shuhan Wen, Jingling Zhang, Peili Wang, Yu Chen, Ping Zhao, Wei Fan, Yanli Zhou, Shengchao Yang, Guangqiang Long

**Affiliations:** ^1^ College of Resources and Environment, Yunnan Agricultural University, KunMing, China; ^2^ The Key Laboratory of Medicinal Plant Biology of Yunnan Province, Yunnan Agricultural University, Kunming, China; ^3^ National & Local Joint Engineering Research Center on Germplasm Innovation & Utilization of Chinese Medicinal Materials in Southwest China, Yunnan Agricultural University, Kunming, China; ^4^ Plant Germplasm and Genomics Center, The Germplasm Bank of Wild Species, Kunming Institute of Botany, Chinese Academy of Sciences, Kunming, Yunnan, China

**Keywords:** cadmium, chicken manure, *E. breviscapus*, remediation, scutellarin

## Abstract

**Introduction:**

Cadmium (Cd) pollution leads to the decrease of the yield and active ingredient content of medicinal plants, and the accumulation of Cd in these plants present potential safety risks for medicinal applications. Exploring effective measures for the safe cultivation of medicinal plants, particularly those with strong capacity for Cd accumulation, is crucial to ensure the safety and quality of medicinal materials.

**Methods:**

In this study, *E. breviscapus*, a medicinal plant with a relatively greater capacity for Cd accumulation, was selected for a pot experiment. The experiment was conducted using soil artificially contaminated with 100 mg kg^−1^ of Cd to evaluate the effects of different application rates of chicken manure (0, 10, 30, and 60 g kg^−1^, designated as CM0, CM10, CM30, and CM60, respectively). The optimal application amount of chicken manure (CM) was determined, and the underlying mechanisms of CM improving the yield and active ingredient contents of *E. breviscapus* was explored from the physiological response of plants and the forms and content of Cd in soil.

**Results:**

The results showed that compared with the CM0, the soil Cd content decreased by 7.0% under CM30 and by 12.3% under CM60. The plant yield increased by 32% in the CM60, while scutellarin content increased by 2.28, 1.92, and 2.72-fold in CM10, CM30, and CM60, respectively. Among all treatments, CM60 demonstrated the most pronounced effect in reducing shoot Cd levels and enhancing both plant yield and scutellarin content. Structural equation modeling (SEM) analysis revealed that the increase of plant yield was primarily attributed to Pn, whereas the enhancement in scutellarin content was associated with shoot Cd concentration and CAT activity in plants.

**Conclusions:**

In summary, this study demonstrates a feasible and environmentally sustainable approach to the safe cultivation of medicinal plants, with the dual benefits of maintaining yield and enhancing active ingredients content.

## 1 Introduction

Cadmium (Cd) poisoning can lead to various human diseases, including renal dysfunction, osteoporosis, multiple types of cancer, and Itai-Itai disease (Román-Ochoa et al., 2023). In particular, when it is absorbed by plants from the soil and then moves through the food chain, this process has received considerable attention (El-Naggar et al., 2020). With exceedance ratios of 38.8%, the 60 medicinal plants’ Cd concentrations were higher above the limits of the “Green Trade Standards of Importing & Exporting Medicinal Plants & Preparations” (Wang et al., 2019). Cadmium pollution leads to the decline of medicinal material output by enhancing antioxidant stress and inhibiting photosynthesis and the nutrient absorption of plants (Wu et al., 2023). Moreover, after Cd enters the medicinal parts of plants, it will increase the risk of being absorbed by the human body and bring about drug safety problems. In addition, strong stress and absorption of Cd into plants may lead to the disorder of secondary metabolites, and such metabolites are critical sources of active ingredients with therapeutic effects in medicinal materials. However, there is relatively limited research on the controls of Cd pollution on medicinal plants.

*Erigeron breviscapus* (Vant.) Hand-Mazz, a traditional Chinese herb, has a therapeutic impact on cerebrovascular and cardiovascular disorders, including ischemic stroke and coronary heart disease (Chen et al., 2022). The therapeutic properties are primarily attributed to flavonoids, such as scutellarin, which is extracted from the shoot of the plant following harvest (Edo et al., 2025). However, previous studies have demonstrated that the over-standard rates of Cd in *E. breviscapus* (limiting value: 0.3 mg kg^-^¹) and in the soil (soil limit value: 0.3 mg kg^-^¹) are as high as 37.0% and 83.3%, respectively. *E. breviscapus* stands out as one of the most common Cd-enriched plants, with an enrichment coefficient of 0.5 (Lu et al., 2018). Because of its widespread use in medicine, ensuring the safety of *E. breviscapus* cultivated in Cd-contaminated environments is vital. Consequently, mitigating the risk of Cd pollution involves not only reducing the bioavailability of Cd in the soil but also minimizing the absorption and translocation of Cd from the roots to the shoot of the plant. This strategy is essential for improving both the quality (defined by an increased content of active ingredients and a reduced Cd content) and yield of *E. breviscapus*, thereby ensuring its efficacy and safety for medicinal applications.

Organic amendments such as manure are increasingly employed as remediation strategies for heavy metal-contaminated soils (Bashir et al., 2021). Among various types of organic amendments, livestock dung is widely used as an organic fertilizer to improve soil health and increase agricultural yields. Studies have shown that replacing mineral fertilizers with organic manure can boost crop yields by 8.5–14.2 Mg ha^−1^ (Chen et al., 2014). Meanwhile, research has demonstrated that organic amendment application can promote the immobilization of Cd in soil, thereby reducing its bioavailability (Yang et al., 2022). A previous study found that the use of chicken manure (CM) significantly reduced soil Cd concentrations compared to control treatments without CM application (Yi et al., 2022). The mechanisms underlying this effect include dissolution, precipitation, and adsorption, through which CM influences the migration and transformation of heavy metals in soil (Zhen et al., 2020). Additionally, the organic matter in CM can bind with metals by adsorption or forming stable organo-metal complexes, thereby reducing their mobility and bioavailability in soil (Halim et al., 2015). While CM has shown promise in improving soil conditions, its impact on the growth performance of medicinal plants and its potential to reduce Cd accumulation remain largely unexplored.

To fill these research gaps, a pot experiment with four application rates of CM on Cd-contaminated soil was conducted to investigate (1) whether the application of CM could improve the yield and active ingredient content and reduce the Cd content in *E. breviscapus*, and (2) how CM application alleviates the stress damage caused by Cd to *E. breviscapus*. Based on previous research, we hypothesize that the appropriate application of CM can significantly improve the growth performance of *E. breviscapus* in Cd-contaminated soil and reduce the stress damage caused by Cd by strengthening the antioxidant defense system of plants and reducing the availability of Cd in soil. Therefore, this study assessed the impact of CM on the growth and Cd stress resistance of *E. breviscapus* by measuring key physiological indicators such as chlorophyll content, antioxidant enzyme activities, mineral element content, and biomass accumulation. Additionally, soil Cd availability and speciation were monitored to elucidate how CM affects the behavior of Cd in the soil–plant system.

## 2 Materials and methods

### 2.1 Experimental materials and design

The pot trials were conducted from May to August 2019 in the Experimental Field of Yunnan Agricultural University in Kunming, Yunnan Province of China (25°13′ N, 102°74′ E). The soil was collected from a 0- to 20-cm soil layer in the experimental field then air-dried under shade and sieved using a 5-mm mesh (original soil). Considering that the concentration of Cd in lead-zinc mining areas in southern China can reach up to 0.26–885.0 mg kg^-^¹, with an arithmetic mean of 114.8 mg kg^-^¹ (Zhang et al., 2023), cultivating conventional crops in such highly Cd-contaminated soils poses significant risks. However, *E. breviscapus*, as a medicinal plant with Cd accumulation capacity, shows certain adaptability to high Cd-contaminated soil. Growing *E. breviscapus* in such high Cd-contaminated soil may provide a feasible strategy for land utilization, helping to avoid the wastage of potentially usable land resources. Therefore, the Cd spiking level should reflect real-world contamination levels while also considering the growth characteristics and tolerance of *E. breviscapus*. Based on this rationale, the original soil was spiked with Cd at a rate of 100 mg kg^-^¹ using CdCl_2_·2.5 H_2_O, and then allowed to stabilize for 1 month before being used in the pot experiment (stabilized soil). The physical and chemical properties of the original soil were as follows: pH value, 6.26; organic matter, 16.37 g kg^−1^; total nitrogen (N), 1.67 g kg^−1^; total phosphorus (P), 1.12 g kg^−1^; total potassium (K), 10.31 g kg^−1^; alkaline hydrolysis N, 132.48 mg kg^−1^; available P, 15.36 mg kg^−1^; available K, 81.86 mg kg^−1^; and total Cd, 2.41 mg kg^−1^.

The CM was purchased from the Fengrun agricultural supermarket with a Cd content of 0.127 mg kg^−1^. Each plastic pot was 22 cm in diameter and 15 cm in height. There were five treatments in these pot trials: CK was filled with 2.5 kg of non-Cd-contaminated soil and no CM addition; CM0 was filled with 2.5 kg of stabilized soil and no CM addition; CM10 was filled with 2.5 kg of stabilized soil and 10 g kg^−1^ CM; CM30 was filled with 2.5 kg of stabilized soil and 30 g kg^−1^ CM; and CM60 was filled with 2.5 kg of stabilized soil and 60 g kg^−1^ CM. The lower dose (e.g., 10 g kg^-^¹) was primarily used to assess whether CM could still effectively improve soil properties and support plant growth even at low application levels. In contrast, the higher doses (e.g., 30 and 60 g kg^-^¹) were selected to explore whether increasing the input of CM would result in a dose-dependent effect on the growth of *E. breviscapus* in Cd-contaminated soil. Furthermore, the choice of these concentrations also referred to the results of our preliminary experiments, ensuring that they could significantly influence soil Cd availability while promoting healthy plant growth without causing excessive nutrient accumulation or other adverse effects.

In order to reduce the experimental deviation, this experiment adopted a completely random design and set up five replicates. A total of 25 potted plants were used. All potted plants were randomly arranged in the greenhouse, and the greenhouse temperature was controlled at 20 ± 5°C to ensure the consistency of environmental conditions among treatments. Specifically, five repeated potted plants for each treatment were evenly distributed throughout the greenhouse to avoid systematic errors caused by position differences. Through this random arrangement, the deviation that may be caused by environmental factors (such as lighting and ventilation) is minimized.

A variety of *E. breviscapus* was the high-quality “Longjin No. 1”, which was provided by Xuanwei Longjin Biotechnology Co., Ltd. (Xuanwei, Yunnan, China). There were 30 seedlings in each pot and the density is consistent with that observed under field cultivation conditions. At the time of seedling transplantation, nitrogen (0.36 g pot^-^¹ as urea), potassium oxide (0.32 g pot^-^¹ as potassium sulfate), and phosphorus pentoxide (0.9 g pot^-^¹ as calcium superphosphate) were added to the soil to supply nutrients for seedling growth (Jia et al., 2023).

Soil and complete *E. breviscapus* plant samples were taken on days 30, 60, and 90. The plant samples were cleaned with deionized water and then divided into two parts: (i) one part was used immediately as fresh samples to determine antioxidant enzyme activity, malondialdehyde (MDA) content, and total chlorophyll content; (ii) another part was oven-dried to constant weight and used as dry samples to determine the content of active ingredients, Cd, and mineral elements. The surface (0–15 cm) soil was collected, naturally air-dried, and sieved (0.15 mm) as soil samples to determine Cd content, available Cd content, and Cd forms in soil.

### 2.2 Measurement of *E. breviscapus* agronomic characters

Agronomic traits including plant height, leaf width, and leaf length were measured *in situ* using a meterstick on days 30, 60, and 90 after transplanting. The yield of *E. breviscapus* (i.e., shoot dry weight) was measured on day 90. During harvest (day 90), 10 representative *E. breviscapus* plants per treatment were randomly selected, carefully uprooted, and rinsed thoroughly with deionized water to remove surface contaminants. The plants were then separated into roots and shoot components, and fresh weights were recorded immediately. Subsequently, these plant samples were oven-dried at 105°C for 30 min and then dried at 75°C until constant weight was achieved. Dry weights of shoots and roots were recorded as yield and root dry weight, respectively.

### 2.3 Measurement of antioxidants of *E. breviscapus*

Fresh leaf tissues (0.5 g) were homogenized in 5 mL of buffer and centrifuged at 12,000 × *g* for 20 min at 4°C. The supernatant was used to determine the activities of peroxidase (POD), catalase (CAT), and superoxide dismutase (SOD) using the corresponding assay kits (Suzhou Gerui Biotechnology Co., Ltd.). MDA content was spectrophotometrically determined following the method applied by Heath and Packer (2022).

### 2.4 Measurement of chlorophyll and photosynthesis

Chlorophyll content was quantified using the ethanol extraction method described by Zhou et al. (2019). Briefly, fresh leaves (0.2 g) were immersed in 10 mL of 95% ethanol in darkness until complete decolorization. Chlorophyll concentration was calculated from absorbance readings at 665 and 649 nm. Net photosynthetic rate (Pn), intercellular CO_2_ concentration (Ci), stomatal conductance (Gs), and transpiration rate (Tr) were measured on fully expanded leaves at 10:00–11:00 a.m. on day 60 using a LI-6400 portable photosynthetic apparatus (LI-COR, Inc., Lincoln, NE, USA) under ambient conditions.

### 2.5 Measurement of foliar mineral elements of *E. breviscapus*

The total concentration (g kg^−1^) of Ca, Mg, Zn, Cu, and Fe in leaf tissues was determined after nitric acid digestion using a graphite furnace atomic absorption spectrometer (GFAAS; Zeenit 65, Analytikjena AG, Jena, Germany).

For nitrogen (N), phosphorus (P), and potassium (K) determination, the dried plant material was first digested using a mixture of concentrated sulfuric acid (H_2_SO_4_) and hydrogen peroxide (H_2_O_2_) and the clear digest was ready for further analysis. Foliar N content was estimated by the Kjeldahl method (Kjeldahl, 1883). Total phosphorus content was quantified using the molybdenum blue colorimetric method with ultraviolet–visible spectrophotometry (UV-Vis, Shimadzu UV-2600, Japan) and potassium concentration was determined using flame photometry (FP, Sherwood Scientific 410C, UK) (Lu, 2000).

### 2.6 Measurements of the active ingredient content of *E. breviscapus*

Active ingredients such as scutellarin, chlorogenic acid, 1,5-di-O-caffeoyl quinic acid, 3,4-di-O-caffeoyl quinic acid, 4,5-di-O-caffeoyl quinic acid, apigenin, and baicalin of *E. breviscapus* leaves were determined by Chinese Pharmacopoeia (National Pharmacopoeia Commission, 2015).

### 2.7 Measurement of Cd content in soil and *E. breviscapus*

Plant Cd content was determined by the HNO_3_-H_2_O_2_ digestion method (Chen et al., 2018). Approximately 0.5 g of finely ground, oven-dried plant samples were digested with concentrated nitric acid (HNO_3_) and hydrogen peroxide (H_2_O_2_). The Cd content of soil samples was determined by HCl-HNO_3_-HCl_4_ (Li and Thornton, 1993). Approximately 0.5 g of soil samples was treated with concentrated hydrochloric acid (HCl), nitric acid (HNO_3_), and perchloric acid (HClO_4_). Both plant and soil extracts were analyzed for Cd concentration using the GFAAS (Zeenit 65, Analytikjena AG, Jena, Germany).

Bioconcentration factor (BCF) and translocation factor (TF) of Cd in different plant partitioning was estimated with the following formula (Hamid et al., 2020):


(1) $$\begin{array}{c} BCF=Root\;Cd\;concentration/Soil\;Cd\;concentration \end{array}$$

(2) $$\begin{array}{c} TF=Shoot\;Cd\;concentration\;/Root\;Cd\;concentration \end{array}$$

### 2.8 Measurement of available Cd and Cd fractions in soil

The soil available Cd was extracted by the DTPA-TEA-CaCl_2_ solution (0.005 mol L^−1^ DTPA, 0.01 mol L^−1^ CaCl_2_, and 0.1 mol L^−1^ TEA, pH 7.3) and analyzed using the GFAAS (Zeenit 65, Analytikjena AG, Jena, Germany) (Lindsay and Norvell, 1978). Soil Cd fractions (exchangeable fraction, reducible fraction, oxidizable fraction, and residual fraction) were extracted using Tessier extraction methods (Tessier et al., 1979). Each fraction was obtained through a series of selective chemical extractions, and the Cd concentration in each was determined by the GFAAS.

### 2.9 Statistical analyses

Shapiro–Wilk normality tests were used to check the normality of the datasets. The differences in soil Cd content, yield, scutellarin, physiological responses, and chemical composition among all treatments were evaluated with one-way analysis of variance (ANOVA). Prior to ANOVA, homogeneity of variance was tested using Levene’s test. For variables that showed significant differences in ANOVA, Duncan’s multiple range test was conducted as a *post-hoc* analysis to determine which specific treatment means differed significantly (*p* < 0.05). Before conducting structural equation modeling (SEM), multicollinearity among variables was assessed using the variance inflation factor (VIF). All variables had VIF values below 10, indicating no serious multicollinearity issues. SEM was built using the Amos 24.0 software package (Small Waters Corporation, Chicago, USA). The adequacy of the models was assessed via chi-square (χ^2^) tests (0 < χ^2^/df ≤ 2), the goodness of fit index (GFI; GFI > 0.9), and the root mean square error of approximation (RMSEA; 0 ≤ RMSEA ≤ 0.05). Principal component analysis (PCA) was employed to evaluate the relationships of shoot Cd content and shoot dry weight with chemical composition under five treatments. The Mantel test was completed in R (version 4.2.1) using the “linket” package, and we performed a one-way ANOVA and the Duncan test to determine significant differences between treatments (*p* < 0.05). Origin 2021 was used to draw graphics.

## 3 Results

### 3.1 Cd content, yield, and active ingredient content in *E. breviscapus*

**Figure 1:**
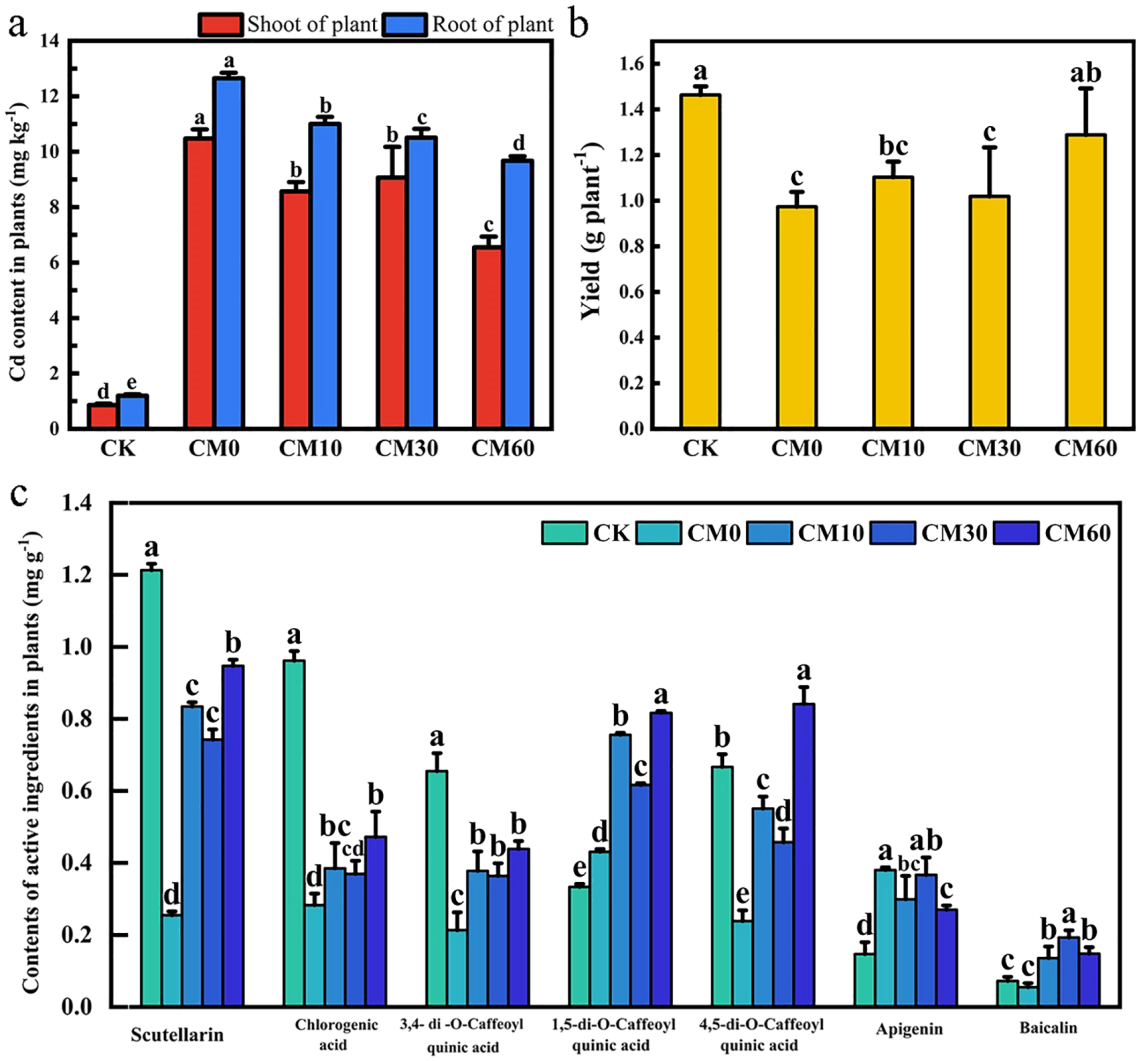
Cd content **(a)**, yield **(b)**, and contents of active ingredients **(c)** in *E breviscapus* under different CM application. CK, no Cd and no CM addition; CM0, 0.01% Cd and 0 g kg^−1^ CM; CM10, 10 g kg^−1^ CM application based on Cd addition; CM30, 30 g kg^−1^ CM application based on Cd addition; CM60, 60 g kg^−1^ CM application based on Cd addition. Different lowercase letters above the error bars indicate significant differences among treatments as revealed by Duncan tests (*p* < 0.05).

Compared with CK, the Cd content of shoots and roots in *E. breviscapus* increased under CM0 (**Figure 1a**). The shoot Cd content is 10.48 mg kg^−1^ and the root Cd content is 12.65 mg kg^−1^ in CM0. The shoot Cd content in CM10, CM30, and CM60 was 8.57, 9.07, and 6.55 mg kg^−1^ and the root Cd content in CM10, CM30, and CM60 was 11.0, 10.51, and 9.67 mg kg^−1^, respectively. Compared with CM0, the shoot Cd content in plant decreased by 18.2%–37.5% and the root Cd content decreased by 13.0%–23.5% in CM10, CM30, and CM60. Compared with CK, plant yield considerably decreased by 57.9% with CM0 (**Figure 1b**). Compared with CM0, there were no significant variations in plant yields between CM10 and CM30, but the yield in CM60 increased by 32.4%. No significant differences were observed in plant yields between CM60 and CK. The active ingredient content decreased in CM0 as compared to CK, except for the contents of 1,5-di-O-Caffeoyl quinic acid, apigenin, and baicalin (**Figure 1c**). The active ingredient content (except apigenin content) of the plant increased under CM10, CM30, and CM60. The scutellarin content of the plant increased by a factor of 2.28, 1.92, and 2.72 in CM10, CM30, and CM60, respectively, compared with CM0. CM60 maintained a lower Cd content in shoots and roots and a higher yield and scutellarin content of plant than CM10 and CM30.

### 3.2 Photosynthetic characteristics and antioxidant enzyme activities of *E. breviscapus*

**Figure 2:**
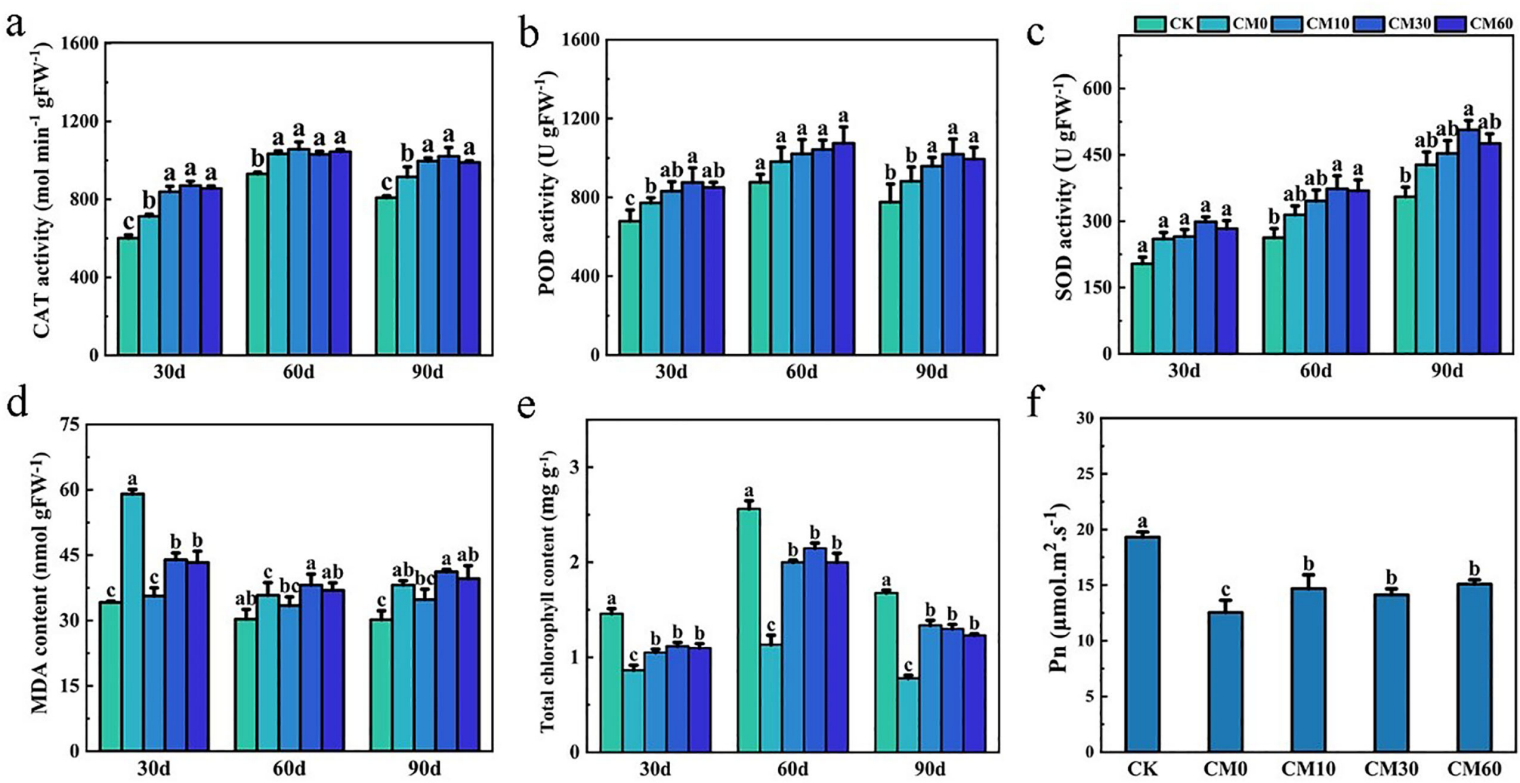
CAT activity **(a)**, POD activity **(b)**, SOD activity **(c)**, MDA content **(d)**, and total chlorophyll content **(e)** on days 30, 60, and 90, and Pn on day 60 **(f)** of *E. breviscapus*. Pn, photosynthetic rate. CK, no Cd and no CM addition; CM0, 0.01% Cd and 0 g kg^−1^ CM; CM10, 10 g kg^−1^ CM application based on Cd addition; CM30, 30 g kg^−1^ CM application based on Cd addition; CM60, 60 g kg^−1^ CM application based on Cd addition. Different lowercase letters above the error bars indicate significant differences among treatments as revealed by Duncan tests (*p* < 0.05).

CAT activity and POD activity of *E. breviscapus* reached the highest values on day 60 under CK, CM10, CM30, and CM60, compared with days 30 and 90 (**Figures 2a, b**). On day 90, CAT activities of plant increased by 8.1%–11.6% and POD activities increased by 8.7%–15.5% under CM10, CM30, and CM60, as compared to CM0. The SOD activity of plants on day 90 was higher than that of plants on days 30 and 60 (**Figure 2c**). When compared with CM0, the MDA content remarkably decreased by 40.0%, 25.6%, and 26.7% in CM10, CM30, and CM60 on day 30, respectively (**Figure 2d**). Compared with CK, total chlorophyll content, Pn, Tr, Gs, and Ci were notably decreased in CM0 on days 30, 60, and 90 (**Figures 2e, f**; **Supplementary Figures 1A, A2**). Relative to CM0, total chlorophyll content increased by 76.4%–89.6%, Pn content increased by 12.5%–20.2%, and Tr content increased by 35.0%–78.0% in CM10, CM30, and CM60.

### 3.3 Mineral element content and plant traits of *E. breviscapus*

**Figure 3:**
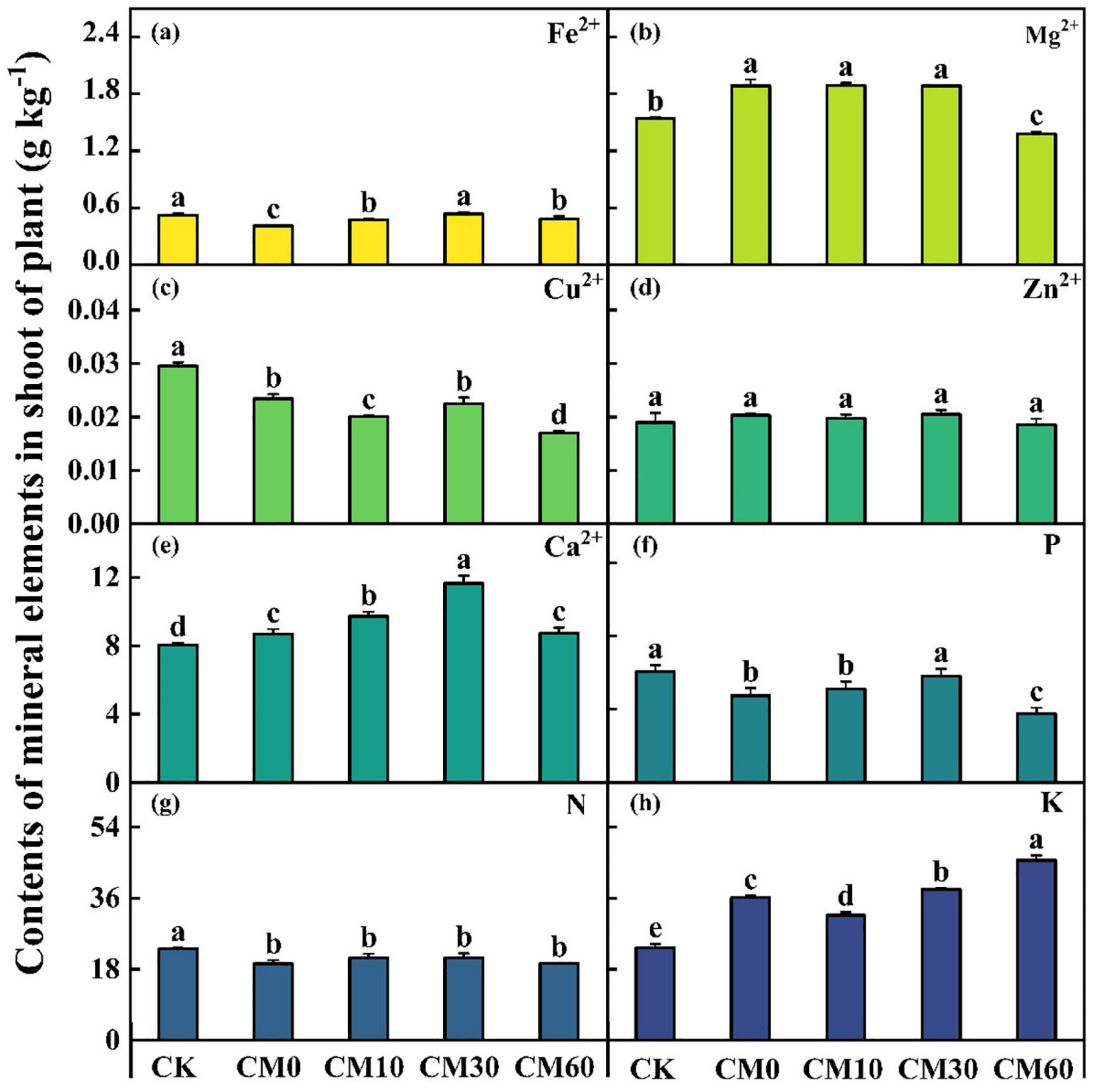
Contents of mineral elements in shoots of *E. breviscapus*. CK, no Cd and no CM addition; CM0, 0.01% Cd and 0 g kg^−1^ CM; CM10, 10 g kg^−1^ CM application based on Cd addition; CM30, 30 g kg^−1^ CM application based on Cd addition; CM60, 60 g kg^−1^ CM application based on Cd addition. Different lowercase letters above the error bars indicate significant differences among treatments as revealed by Duncan tests (*p* < 0.05).

A decrease in the concentrations of Fe²^+^, Cu²^+^, P, and N was observed under Cd addition (CM0), whereas the concentrations of Mg²^+^, Ca²^+^, and K in the shoots of *E. breviscapus* increased, compared with the control (CK) (**Figure 3**). Compared with CK, Cd addition (CM0) led to a decrease in the contents of Fe^2+^, Cu^2+^, P, and N, while the contents of Mg^2+^, Ca^2+^, and K in the shoot of *E. breviscapus* increased (**Figure 3**). Among CM10, CM30, and CM60, CM30 demonstrated a relative advantage in terms of the increase in mineral element content in plant shoots. The content of K in plant shoots increased by 26.1% under CM60 compared with CM0. The fresh weight in shoots and roots, the dry weight in roots, and the plant height of *E. breviscapus* remarkably decreased in CM0 compared with CK, but those in CM10, CM30, and CM60 remarkably increased as compared to CM0 (**Supplementary Table A1**). Additionally, the maximum measured values of the fresh weight of shoots and roots, and plant height were found in CM60.

### 3.4 Content of Cd, available Cd, and Cd fractions in soil

**Figure 4:**
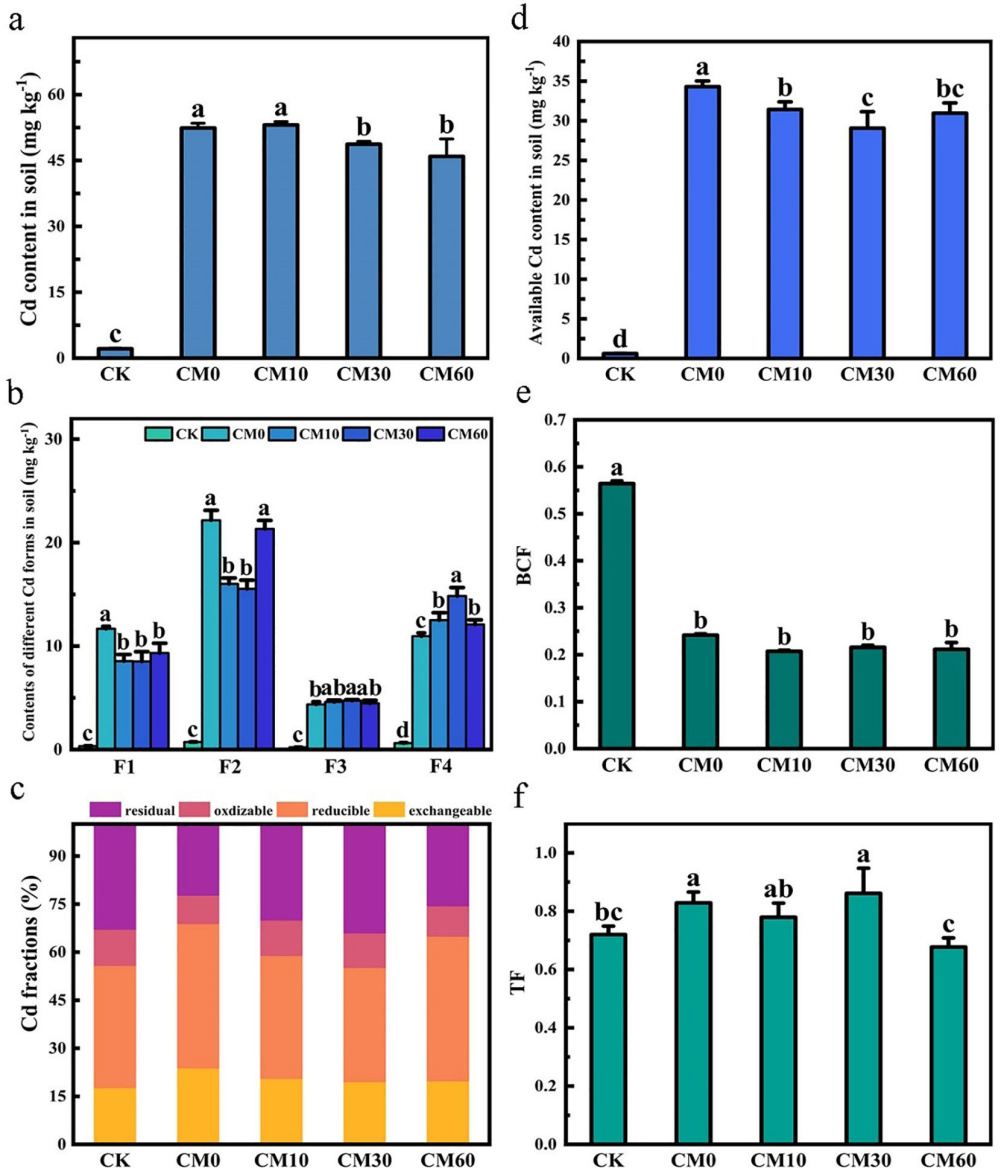
Soil Cd content **(a)**, content of different Cd forms **(b)**, Cd fractions **(c)**, available Cd content in soil **(d)**, BCF **(e)**, and TF **(f)** of *E. breviscapus*. F1, exchangeable Cd; F2, reducible Cd; F3, oxidizable Cd; F4, residual Cd. BCF, bioconcentration factor; TF, translocation factor. CK, no Cd and no CM addition; CM0, 0.01% Cd and 0 g kg^−1^ CM; CM10, 10 g kg^−1^ CM application based on Cd addition; CM30, 30 g kg^−1^ CM application based on Cd addition; CM60, 60 g kg^−1^ CM application based on Cd addition. Different lowercase letters above the error bars indicate significant differences among treatments as revealed by Duncan tests (*p* < 0.05).

In comparison to CM0, the soil Cd content on day 90 when the *E. breviscapus* were harvested was decreased by 7.0% and 12.3% in CM30 and CM60, respectively (**Figure 4a**). Compared with CM0, the exchangeable Cd content decreased by 26.9%, 27.1%, and 20.1% and the residual Cd content increased by 18.9%, 22.6%, and 18.3% in CM10, CM30, and CM60, respectively (**Figures 4b, c**). Reducible Cd content decreased by 27.8% and 30.0% in CM10 and CM30 relative to CM0. The available Cd content in soil decreased by 8.4% and 15.3% in CM30 and CM60, respectively (**Figure 4d**). There were no significant variations in Equation 1 between CM0, CM10, CM30, and CM60 (**Figure 4e**). However, the Equation 2 in CM60 significantly decreased compared with that in CM0, CM10, and CM30 (**Figure 4f**).

### 3.5 Link between soil Cd content, yield, and scutellarin of *E. breviscapus*

**Figure 5:**
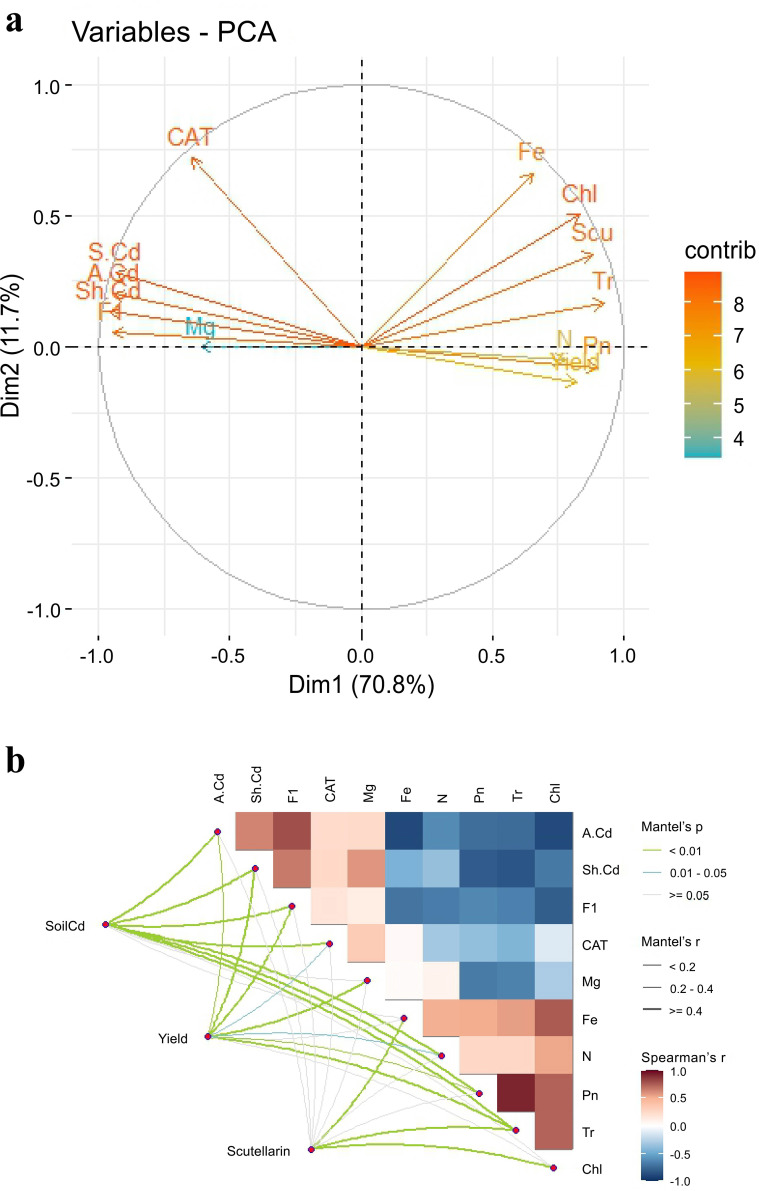
PCA **(a)** of physiological and biochemical parameters in soil and plants under CM addition. Correlations between soil Cd content, yield, and scutellarin content of *E. breviscapus* with physiological and biochemical parameters **(b)**. Colors indicate correlation types. Line width corresponds to the partial Mantel’s *r* statistic. Physiological and biochemical parameters are also shown with a color gradient denoting Pearson’s correlation coefficient. The variables included the following: Scu, scutellarin content of shoot; Sh. Cd, content of Cd in shoot of plant; **(a)** Cd, available Cd content in soil; Chl, chlorophyll content in plant, Pn, photosynthetic rate of plant; Tr, transpiration rate of plant; F1, exchangeable Cd content in soil.

**Figure 6:**
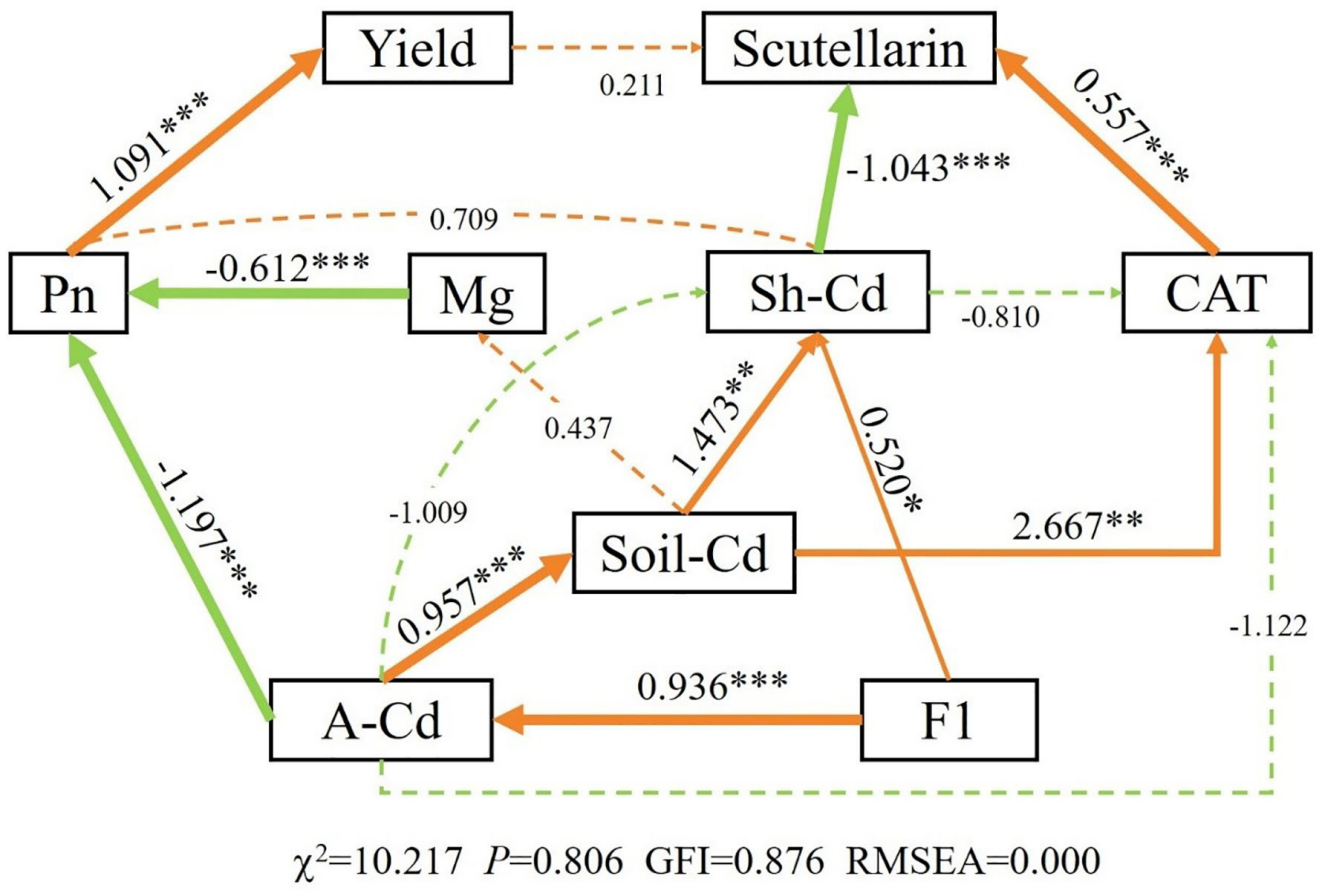
Structural equation modeling (SEM) showing the direct and indirect effects of soil Cd content, yield, and scutellarin in *E. breviscapus*. Orange arrows represent positive relationship, and green arrows represent negative relationship. The number next to the arrows are standardized path coefficients. The width of the arrow line indicates the strength of the relationship. The dashed arrows represent nonsignificant (*p* > 0.1) relationships. ***p < 0.001; **p < 0.01; *p < 0.05. A-Cd, available Cd content in soil; Sh-Cd, shoot Cd content in plant; F1, exchangeable Cd content in soil; Scu, the scutellarin content in plant; Pn, photosynthetic rate of plant; CAT, catalase activity of plant; Mg, Mg^2+^ content of plant leaves.

The results of PCA indicated that PC1 and PC2 explained 82.5% of the overall variability in the data, implying that different applications of CM induced a significant influence (**Figure 5a**). The soil Cd content (S. Cd) was positively correlated with the available Cd content (A. Cd) and the exchangeable Cd content (F1) in soil, yield was positively correlated with N and Pn of *E. breviscapus*, and scutellarin (Scu) was positively correlated with chlorophyll (Chl). SEM suggested that Pn, CAT, and available Cd exhibited direct positive impacts on yield, scutellarin content, and soil Cd content, respectively (**Figure 6**). The Mantel test showed that scutellarin had a positive correlation with chlorophyll, Fe, and Tr (**Figure 5b**). The Cd content of shoots (Sh. Cd) was negatively correlated with yield and scutellarin, but positively correlated with soil Cd content.

## 4 Discussion

### 4.1 Yield of *E. breviscapus* was increased under sufficient CM application

Compared with CM0, no significant differences were observed in the yield of CM10 and CM30, but the yield in CM60 was increased by 32.0%. However, when compared with CK, CM60 showed no significant influence on the yield, which indicated that the yield of *E. breviscapus* in high-concentration Cd-contaminated soil can be improved by sufficient CM (**Figure 1b**). A previous study showed that livestock manure can reduce soil nutrient loss, improve soil fertility, and promote plant biomass, thus increasing crop yield (Li et al., 2021). Cd is classified as non-essential for plant metabolic reactions and causes a significant reduction in plant development and yield, even at the least amount (Zia-ur-Rehman et al., 2023). In this study, Cd content in plant shoots and roots decreased under CM10, CM30, and CM60 compared with CM0 (**Figure 1a**). The reduction of Cd content in plants may alleviate the direct damage caused by Cd to plants and promote the increase in plant yield. Additionally, a significant negative correlation was observed between yield and shoot Cd content in *E. breviscapus* (**Figure 5a**).

Chlorophyll was shown to spontaneously form a complex with cadmium (Cd-Chl), which is incorporated at the central position of the chlorophyll molecule porphyrin ring, where it replaces Mg. The drop in nonradiative energy transfer will occur between molecules in the system in which Cd-substituted chlorophyll will appear, which will cause a significant decrease in photosynthesis effectiveness (Grajek et al., 2020). In this study, total chlorophyll content and Pn were increased in CM10, CM30, and CM60 compared with CM0 (**Figures 2e, f**). The decrease of the Cd content in plants may decrease the Cd-Chl complex, resulting in the increase of total chlorophyll content and Pn. Additionally, Pn showed a positive correlation with yield; thus, the increase in Pn may improve plant yield (**Figure 6**).

### 4.2 Addition of CM promoted the increase of scutellarin content in *E. breviscapus*

Under Cd stress conditions, plants can accumulate secondary metabolites to prevent oxidative damage caused by Cd (Okem et al., 2015). However, in this work, the content of most secondary metabolites of *E. breviscapus* was notably decreased, caused by the high concentration of Cd (**Figure 1c**). Hence, Cd with a high concentration will reduce the accumulation of secondary metabolites in plants. A previous study found that the Cd concentration in grain is mainly related to the bioavailability of Cd in soil rather than the total Cd content and the potential toxicity of heavy metals is related to their bioavailability and chemical speciation (Kim et al., 2016). In this study, SEM showed that exchangeable Cd content positively correlated with shoot Cd content in plants (**Figure 6**). Synchronously, compared with CM0, the content of lower-bioavailability residual Cd increased 18.3%–22.6% and the higher-bioavailability exchangeable Cd decreased 20.1%–27.1%; the available Cd content in soil decreased 7.0%–15.3% in CM10, CM30, and CM30 (**Figures 4b, d**) (Huang et al., 2016). This study indicated that the application of CM could promote the transformation from exchangeable Cd to residual Cd, thereby reducing the content of Cd in plants. At this time, Cd concentration in plants may promote the production of plant secondary metabolites. Therefore, the reduction of Cd content in shoots also promoted the production of scutellarin, thus improving the quality of *E. breviscapus*, and SEM also illustrated that shoot Cd content has a negative correlation with scutellarin (**Figure 6**).

One of the earliest effects of plant cells being exposed to toxic concentrations of heavy metals is the production of reactive oxygen species (ROS), i.e., superoxide (O•− 2) and hydroxyl radicals (^•^OH), as well as non-radicals, such as hydrogen peroxide (H_2_O_2_) and singlet oxygen (^1^O_2_) (Berni et al., 2019). Plants evolved enzymatic machinery to protect against the toxic effect of ROS and maintain their cellular redox homeostasis (Petrov et al., 2015). The mechanism relies on enzymes such as SOD, POD, and CAT. This study showed that the activities of POD, SOD, and CAT were enhanced and MDA content was remarkably decreased in CM10, CM30, and CM60, when compared with CM0 (**Figure 2**). PCA and Mantel analysis showed a positive correlation between CAT and shoot Cd content (**Figure 5**). Additionally, CAT positively correlated with scutellarin content (**Figure 6**). These findings revealed that the application of CM could increase the antioxidant enzymes’ activities, thereby alleviating the stress of Cd on plants and augmenting scutellarin content in *E. breviscapus*.

### 4.3 Application of CM reduced the Cd content in *E. breviscapus*

Compared to CM0, the application of CM reduced Cd concentrations in both the shoots and roots of *E. breviscapus*, by 18.2%–37.5% and 3.0%–23.5%, respectively. Although the BCF (Equation 1) has no significant differences between CM0, CM10, CM30, and CM60, the total Cd content in the soil was reduced by 8.4% and 15.3% under CM30 and CM60 treatments, respectively, leading to a corresponding reduction in root Cd uptake. Furthermore, when CM was applied at the highest rate (60 g kg^-^¹, CM60), the TF (Equation 2) was significantly decreased, indicating a reduced capacity of Cd to migrate from roots to shoots in *E. breviscapus*. According to relevant research, the application of CM in hemp effectively reduces the accumulation of Cd in the plant (Sangsoda et al., 2025). Consequently, shoot Cd concentration in the CM60 treatment was significantly lower than that in CM10 and CM30. In addition, changes in Cd speciation in the soil may also influence Cd uptake by *E. breviscapus*. The application of CM could alter Cd fractions in the soil, reducing its bioavailability and thus further alleviating Cd toxicity to the plant. Available Cd content in soil decreased by 8.4% and 15.3% in CM30 and CM60, respectively (**Figure 4d**). Soil pH is closely related to the solubility of heavy metals. CM can significantly increase the pH value of the soil. The enhancement of pH increases the number of negative charges on the surface of soil substrates and organic matter, thereby enhancing their binding capacity for cations, which could reduce the bioavailability of Cd (Yu et al., 2024). The addition of CM reduced the acid-extractable Cd content, increased the residual Cd content, and decreased the total Cd content in the soil (**Figure 4**). The increase in stable Cd content in this study indicates that CM significantly passivates Cd, reducing its toxicity. These findings are consistent with those of previous studies (Rehman et al., 2023). The organic matter in CM can immobilize metals by adsorption or forming stable organo-metal complexes, thereby reducing their mobility and bioavailability in soil (Halim et al., 2015).

Based on our previous investigations, the utilization of CM exhibited superiority in enhancing crop yield and the accumulation of active compounds compared to biochar, lime, and hydroxyapatite (Jia et al., 2024; Liu et al., 2024; Zhang et al., 2023). This advantage is especially pronounced when considering factors such as availability and cost-effectiveness. Therefore, CM can serve as an ideal passivating agent to effectively alleviate Cd-induced stress in *E. breviscapus*, while simultaneously enhancing both its biomass yield and scutellarin content.

Overall, the application of CM not only significantly enhances the biomass and active ingredient content of *E. breviscapus*, but also effectively reduces Cd accumulation in the plant, indicating its promising potential in mitigating heavy metal stress and ensuring both the yield and quality of medicinal plants under Cd contamination. CM60 proved to be better in decreasing soil Cd content, promoting the yield and quality of *E. breviscapus*. In addition, we still need to pay attention to the amount of livestock manure added. Only a small amount of heavy metals in feeds can be absorbed and utilized by livestock, and most of them are discharged through manure (Chen et al., 2022). Therefore, excessive application of livestock and poultry manure could lead to heavy metal pollution of agricultural soils (Liu et al., 2020). Future research should be based on long-term field experiments, and such research should be carried out in different soil environments to determine a better application concentration.

## 5 Conclusion

The optimum dose of CM was 60 mg kg^−1^, which reduced the shoot Cd content and increased the yield and quality of *E. breviscapus*. Furthermore, CM application increased antioxidant enzyme activities, photosynthesis, and chlorophyll content, and reduced the available Cd content in soil. The physiological response of *E. breviscapus* and the change in soil Cd forms alleviated the stress of high Cd concentration on plants and improved the yield and active ingredient content of *E. breviscapus*. Summing up, this study strongly demonstrated that adequate application of CM could relieve the damage caused by Cd to plants and promote the quality of *E. breviscapus*.

## Data Availability

The raw data supporting the conclusions of this article will be made available by the authors, without undue reservation.
