# Supplementary material for: Adequate application of chicken manure could relieve the damage caused by Cd to E. breviscapus plants
Source: Front Plant Sci. 2025 Sep 4;16:1539907. doi: 10.3389/fpls.2025.1539907 (PMC12445149; doi:10.3389/fpls.2025.1539907)
Supplement: Supplementary file 1 [file DataSheet1.docx]

Table A1 Plant fresh weight, root dry weight plant height, leaves length and leaves width of *E. breviscapus*.

| Treatment | Fresh weight  (g plant^-1^) | | Root dry weight  (g plant^-1^) | Plant  height (cm) | Leaves  length (cm) | Leaves width (cm) |
| --- | --- | --- | --- | --- | --- | --- |
|  | Shoot | Root |  |  |  |  |
| CK | 9.19±0.81b | 1.96±0.12a | 0.36±0.04a | 20.41±0.66a | 21.96±0.43a | 2.79±0.14c |
| CM0 | 6.42±0.48d | 0.92±c0.02d | 0.15±0.02c | 16.64±0.29c | 21.33±2.90a | 2.60±0.18bc |
| CM10 | 7.70±0.20bc | 1.36±0.19b | 0.23±0.04b | 17.03±0.32c | 22.40±1.55a | 3.11±0.18ab |
| CM30 | 7.54±1.71bc | 1.11±0.12c | 0.19±0.03bc | 18.05±0.38b | 24.83±1.21a | 3.28±0.46a |
| CM60 | 10.09±1.35a | 1.43±0.06b | 0.21±0.01b | 18.32±0.67b | 22.40±3.06a | 2.99±0.40bc |

Note: CK, no Cd and no CM addition; CM0, 0.01% Cd and 0 g kg^-1^ CM; CM10, 10 g kg ^-1^ CM application based on Cd addition; CM30, 30 g kg^-1^ CM application based on Cd addition; CM60, 60 g kg^-1^ CM application based on Cd addition. Data are average of three replicates ±SE. The different letters in the same column mean a significant difference at *P* < 0.05.


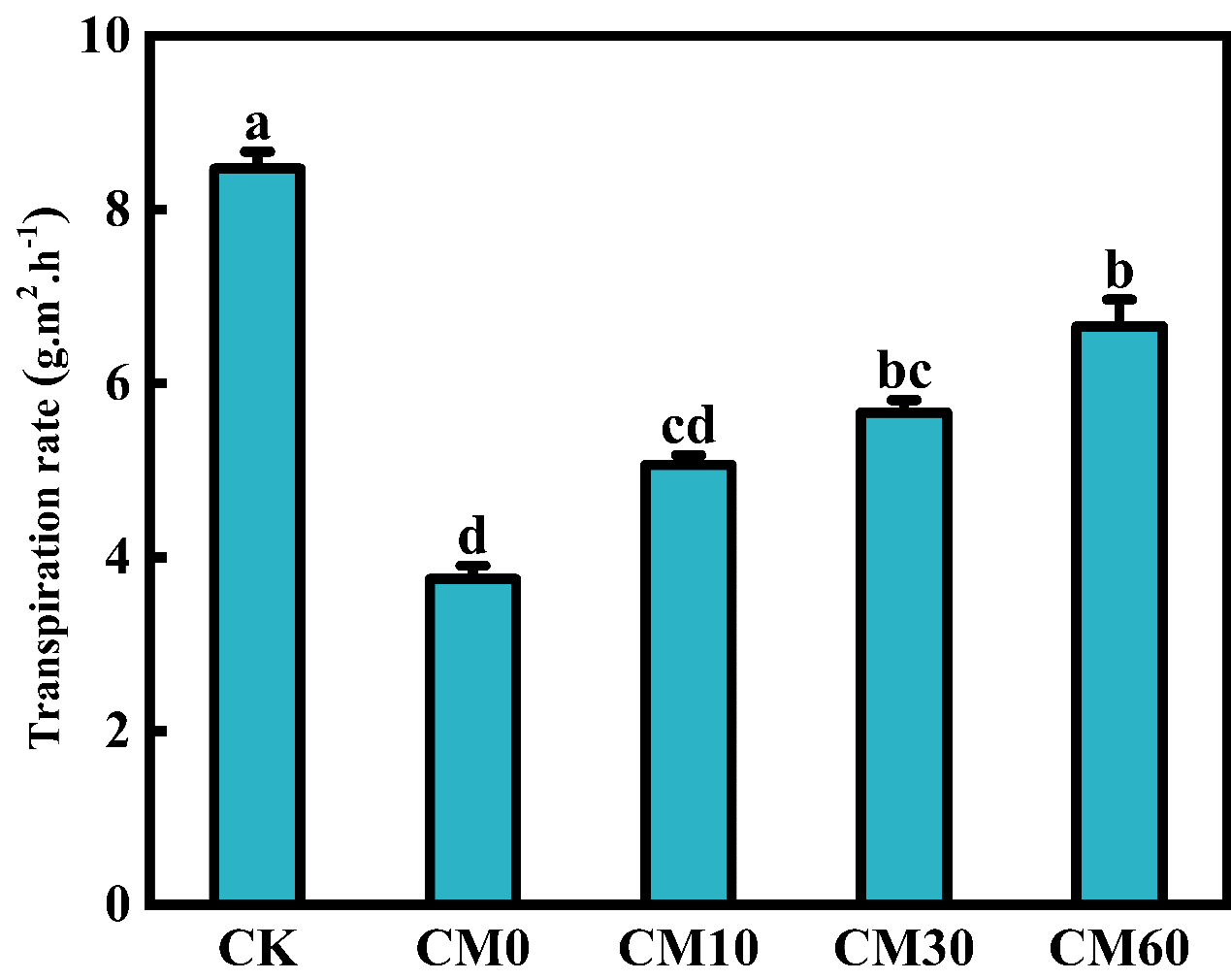


Fig. A1 Transpiration rate of *E. breviscapus.* CK, no Cd and no CM addition; CM0, 0.01% Cd and 0 g kg^-1^ CM; CM10, 10 g kg ^-1^ CM application based on Cd addition; CM30, 30 g kg^-1^ CM application based on Cd addition; CM60, 60 g kg^-1^ CM application based on Cd addition. Different lowercase letters above the error bars indicate significant differences among treatments as revealed by Duncan tests (*P* < 0.05).


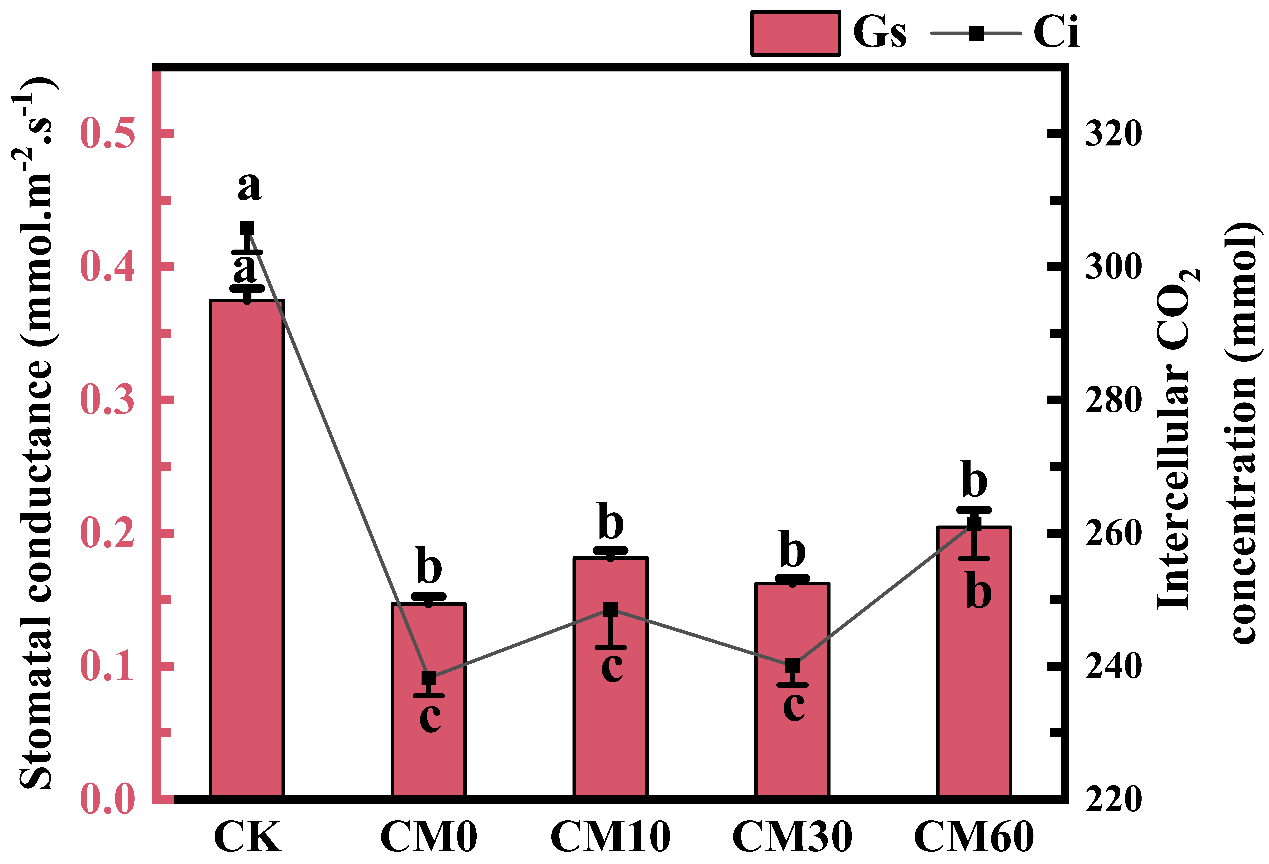


Fig. A2 Stomatal conductance and intercellular concentration of *E. breviscapus*. CK, no Cd and no CM addition; CM0, 0.01% Cd and 0 g kg^-1^ CM; CM10, 10 g kg ^-1^ CM application based on Cd addition; CM30, 30 g kg^-1^ CM application based on Cd addition; CM60, 60 g kg^-1^ CM application based on Cd addition. Different lowercase letters above the error bars indicate significant differences among treatments as revealed by Duncan tests (*P* < 0.05).
